# Efficacy and Safety of Accelerated Transepithelial Corneal Crosslinking in Non-Pediatric Patients with Progressive Keratoconus: Insights from a Retrospective Cohort Study

**DOI:** 10.3390/healthcare13050567

**Published:** 2025-03-06

**Authors:** Alina-Cristina Chiraples, Mihnea Munteanu, Horia T. Stanca, Diana-Maria Darabus, Diana Barakat, Alina-Gabriela Negru

**Affiliations:** 1 Department of Ophthalmology, “Victor Babes” University of Medicine and Pharmacy, 300041 Timisoara, Romania; alina.chiraples@umft.ro (A.-C.C.); diana.darabus@umft.ro (D.-M.D.); 2 Oftalmo Sensory-Tumor Research Center-ORL (EYE-ENT), 300041 Timisoara, Romania; 3 Department of Ophthalmology, “Carol Davila” University of Medicine and Pharmacy, 050474 Bucharest, Romania; horia.stanca@umfcd.ro; 4 Department of Ophthalmology, Timisoara Emergency Clinical Municipal Hospital, 300041 Timisoara, Romania; barakatdiana96@gmail.com; 5 Department of Cardiology, University of Medicine and Pharmacy, 300041 Timișoara, Romania; alinanegru@umft.ro; 6 Department of Cardiology, Institute of Cardiovascular Diseases, 300310 Timisoara, Romania

**Keywords:** transepithelial accelerated corneal crosslinking, progressive keratoconus, visual acuity, corneal tomography, refractive stability, pachymetry, keratometry

## Abstract

Background/Objectives: Transepithelial accelerated corneal crosslinking (TE-ACXL) is a minimally invasive approach for stabilizing progressive keratoconus while preserving the corneal epithelium. This study aims to evaluate changes in visual acuity, refractive error, and corneal parameters before and six months after TE-ACXL. Methods: A retrospective analysis was conducted on 30 eyes from 20 patients who underwent TE-ACXL between May 2021 and June 2023. Variables included were uncorrected distance visual acuity (UDVA) and corrected distance visual acuity (CDVA), spherical and cylindrical refractive error, and corneal tomography parameters such as maximum keratometry (Kmax), the symmetry index (Si), the keratoconus vertex (KV), Baiocchi–Calossi–Versaci index (BCV), thinnest corneal thickness (TCT), and central corneal thickness (CCT). Results: CDVA improved from 0.20 ± 0.22 to 0.06 ± 0.11 LogMAR (*p* = 0.004), while UDVA improved from 0.47 ± 0.35 to 0.29 ± 0.30 LogMAR (*p* < 0.001). Spherical and cylindrical refractive error showed significant reductions from −2.18 ± 3.05 D to −1.31 ± 1.84 D (*p* < 0.001) and −3.33 ± 1.98 D to −2.33 ± 1.52 D (*p* < 0.001), respectively. Pachymetry values decreased significantly, with TCT reducing from 466.43 ± 31.24 µm to 438.63 ± 30.54 µm (*p* < 0.001) and CCT from 480.80 ± 33.24 µm to 451.23 ± 29.26 µm (*p* < 0.001). Kmax showed a modest reduction (52.33 ± 3.51 D to 51.19 ± 3.63 D, *p* < 0.001), while other topographic indices, including Si, KV, and BCV, exhibited minor, non-significant changes, except for BCV back (*p* = 0.031). Conclusions: TE-ACXL was associated with significant improvements in visual acuity and refractive stability at six months postoperatively while maintaining a favorable safety profile. The procedure may serve as an effective option for early intervention in progressive keratoconus.

## 1. Introduction

Keratoconus (KC) is a progressive, bilateral, and asymmetric corneal ectatic disorder characterized by progressive thinning and steepening of the cornea, leading to irregular astigmatism and visual impairment with a reported prevalence ranging from 50 to 600 per 100,000 people, depending on factors such as ethnicity, geographic location, and diagnostic criteria [1]. The disease is associated with both genetic predisposition and environmental factors, such as excessive eye rubbing and nocturnal ocular compression [2]. Epidemiological data indicate wide variations in prevalence rates globally, with higher rates reported in regions such as the Middle East and South Asia [3]. If left untreated, KC can lead to significant deterioration in visual acuity, necessitating invasive procedures such as corneal transplantation [4]. Keratoconus presents distinct characteristics in pediatric and non-pediatric populations. Vilares-Morgado et al. [2] noted that pediatric keratoconus tends to progress more aggressively, often requiring keratoplasty at an earlier stage. Similarly, Oliverio et al. [5] reported that younger patients exhibit a higher likelihood of disease progression despite treatment, underscoring the challenges of stabilizing keratoconus in this subgroup.

The management of keratoconus varies depending on disease severity. In early stages, spectacles and contact lenses, including rigid gas-permeable lenses, can provide sufficient optical correction [6]. However, these options do not address disease progression. Corneal collagen cross-linking (CXL) has emerged as the only minimally invasive intervention capable of halting KC progression by biomechanically strengthening the corneal stroma through photochemical induction of cross-links between collagen fibrils using riboflavin and ultraviolet-A (UV-A) irradiation [6]. Since its introduction, the standard Dresden protocol (epi-off CXL) has been widely adopted and has demonstrated long-term efficacy in stabilizing KC and reducing the need for corneal transplantation [7]. However, the necessity of epithelial debridement in the conventional protocol results in postoperative pain, increased risk of microbial infections, corneal haze, and prolonged recovery time [8].

To mitigate these complications, transepithelial corneal cross-linking (TE-CXL or epi-on) was developed as an alternative approach that preserves the corneal epithelium, theoretically reducing patient discomfort and the risk of infection [9]. However, early studies reported suboptimal efficacy due to the limited penetration of riboflavin through the intact epithelium and reduced oxygen availability required for the photochemical reaction [10]. Various strategies have since been implemented to enhance riboflavin diffusion, including the addition of permeability enhancers such as ethylenediaminetetraacetic acid (EDTA), benzalkonium chloride (BAC), and trometamol (Tris) [2]. Additionally, iontophoresis-assisted riboflavin delivery and modifications in UV-A exposure patterns have been explored to optimize the efficacy of TE-CXL [9].

Building upon these advancements, transepithelial accelerated cross-linking (TE-ACXL) has emerged as a promising alternative that reduces overall treatment time while maintaining sufficient cross-linking efficacy. This approach relies on increased UV-A fluence delivered over a shorter duration, adhering to the Bunsen–Roscoe law of reciprocity [6]. While accelerated protocols have demonstrated comparable stabilization to conventional CXL in some studies, concerns remain regarding the oxygen diffusion limitations and biomechanical effectiveness of TE-ACXL [9,11]. Moreover, most studies investigating accelerated CXL have focused on pediatric populations due to the more aggressive disease course in younger patients, leaving a gap in the literature regarding its effectiveness in non-pediatric patients [4].

Given these considerations, the present study aims to evaluate the efficacy and safety of TE-ACXL in non-pediatric patients with progressive keratoconus. By analyzing clinical and tomographic outcomes in a retrospective cohort, this study seeks to contribute real-world evidence on the viability of TE-ACXL as an alternative to conventional CXL for keratoconus stabilization.

## 2. Materials and Methods

### 2.1. Design

This study is a retrospective cohort analysis aimed at assessing the efficacy and safety of TE-ACXL in adults with progressive KC. It includes patients treated at the Prof. Munteanu Ophthalmologic Center, part of the “Victor Babeş” University of Medicine and Pharmacy in Timişoara, Romania, between May 2021 and June 2023.

This study was conducted in accordance with the principles outlined in the Declaration of Helsinki for research involving human subjects. Given its retrospective design, which involved the analysis of pre-existing clinical data, the requirement for ethical review and approval was waived by the Ethics Committee of “Victor Babeş” University of Medicine and Pharmacy. The study did not involve any direct patient contact, new data collection, or interventional procedures, ensuring that there was no additional risk to patient welfare.

To maintain patient confidentiality, all data were fully anonymized before analysis, with no identifying information linked to the dataset. Digital informed consent was obtained from all participants at their initial clinical visit, explicitly allowing their anonymized medical records to be used for academic and research purposes. The consent process outlined the study’s objectives, procedures, and scope, ensuring that participants were fully informed about the use of their data. This retrospective approval enabled the inclusion of historical clinical records in the present study.

### 2.2. Inclusion and Exclusion Criteria

Patients included in this study were those who underwent TE-ACXL and had a minimum follow-up period of six months. The selection criteria aimed to ensure that only cases with sufficient post-treatment data were analyzed, allowing for a reliable assessment of clinical outcomes. Inclusion criteria were (1) age 18 years or older at the time of treatment, (2) diagnosis of progressive KC, defined as an increase in Kmax of at least 1 diopter over a 12-month period, and (3) pre-treatment central corneal thickness (CCT) of at least 400 µm to ensure safe application of UV-A irradiation. Exclusion criteria were (1) follow-up shorter than six months, (2) presence of other ocular conditions, such as retinal pathology, significant corneal scarring, or severe dry eye disease, that could interfere with the interpretation of outcomes, (3) history of ocular infections or inflammatory diseases in the preceding six months, (4) history of corneal or intraocular surgery, including corneal transplantation, cataract surgery, or lens replacement, in the six months prior to treatment, and (5) use of alternative surgical treatments for KC during the study period.

### 2.3. Surgical Technique

All patients underwent TE-ACXL following a standardized protocol at Prof. Munteanu Ophthalmologic Center. The procedure was performed in a sterile operating environment under topical anesthesia to ensure patient comfort. Preoperatively, patients received oxybuprocaine hydrochloride 4 mg/mL eye drops (Alcaine^®^, Alcon Laboratories, Fort Worth, TX, USA), instilled three times at five-minute intervals. This ensured adequate corneal anesthesia while avoiding excessive epithelial disruption. The riboflavin solution used was a 0.25% riboflavin HPMC formulation containing BAC, EDTA, and TRIS (ParaCel^®^, Avedro Inc., Waltham, MA, USA). This formulation was specifically designed to facilitate stromal penetration without requiring epithelial debridement. Riboflavin instillation was performed over a 15 min period, with the solution applied every two minutes to ensure uniform absorption. The presence of riboflavin saturation in the corneal stroma was confirmed by observing a yellow flare under slit-lamp examination. If necessary, additional instillation was performed to achieve adequate riboflavin diffusion before UV-A exposure.

Following successful riboflavin saturation, the cornea was irradiated using the Avedro KXL system (Avedro Inc., Waltham, MA, USA), which delivered pulsed UV-A light at 45 mW/cm² for 5 min and 20 s, resulting in a total energy dose of 7.2 J/cm². The pulsed 1:1 duty cycle was selected to optimize oxygen diffusion and enhance cross-linking efficacy. Throughout the irradiation phase, Paracel solution (riboflavin part 2) was instilled every 15 s to maintain adequate corneal hydration and UV absorption. At the end of the procedure, the cornea was thoroughly rinsed with balanced salt solution (BSS, Alcon Laboratories, Fort Worth, TX, USA), followed by the instillation of moxifloxacin 0.5% antibiotic drops (Vigamox^®^, Alcon Laboratories, Fort Worth, TX, USA). A bandage contact lens (PureVision^®^, Bausch & Lomb, Rochester, NY, USA) was placed on the treated eye to enhance post-operative comfort and protect the corneal surface during initial healing. Patients were instructed to use topical antibiotics (Vigamox^®^, Alcon Laboratories, Fort Worth, TX, USA) for one week, preservative-free artificial tears for four weeks (Thealoz Duo^®^, Théa Pharmaceuticals, Clermont-Ferrand, France), and fluorometholone 0.1% eye drops (FML^®^, Allergan, Irvine, CA, USA) three times daily for three weeks, starting one week after treatment. Patients were instructed to use the prescribed postoperative eye drops for a duration of three weeks following the procedure. Beyond this period, continued use of eye drops was not required by the study protocol. Any additional use of lubricants or other ophthalmic treatments was at the discretion of the treating clinician and was not systematically recorded.

### 2.4. Measurements

To evaluate the efficacy of TE-ACXL, a series of visual acuity and corneal tomography parameters were assessed before the procedure and at the six-month follow-up. Uncorrected distance visual acuity (UDVA) and corrected distance visual acuity (CDVA) were measured using a Snellen eye chart, following the European standard EN ISO 8596. Visual acuity values were recorded in decimal and LogMAR notation for consistency in analysis. All corneal tomography measurements were obtained using the Sirius^®^ Scheimpflug-based corneal tomography system (CSO Inc., Florence, Italy), which provides a comprehensive keratoconus evaluation report. The following parameters were analyzed: (1) maximum corneal curvature (Kmax): this represents the steepest point of the cornea, commonly used to monitor keratoconus progression, (2) central corneal thickness (CCT) and thinnest corneal thickness (TCT): these measurements were used to assess structural changes in the corneal stroma, (3) symmetry index of the front and back cornea (Sif and Sib): these indices evaluate the degree of vertical asymmetry by comparing curvature differences in the superior and inferior corneal hemispheres, (4) keratoconus vertex front and back (KVf and KVb): defined as the highest points of ectasia on the anterior and posterior elevation maps, and (5) Baiocchi–Calossi–Versaci index (BCVf and BCVb): evaluates the presence and severity of corneal ectasia by analyzing coma and trefoil aberrations based on Zernike decomposition.

### 2.5. Statistical Analysis

All statistical analyses were conducted using IBM SPSS Statistics for Windows, version 29.0 (IBM Corp., Armonk, NY, USA). Data were presented as mean ± standard deviation (SD), median (interquartile range), or frequencies, as appropriate. A sample size calculation was performed to determine the minimum number of eyes required to detect a significant difference in corneal tomography and visual acuity outcomes. The sample size was calculated based on a power analysis with an α level of 0.05, 80% power, an estimated standard deviation of 3.0 D, and a minimum detectable difference of 2.0 D in Kmax [9,12,13,14,15]. This determined the required size of 25 eyes. Since 30 eyes were included, the sample size was considered sufficient for statistical comparisons.

The Shapiro–Wilk test was used to assess the normality of continuous variables. Depending on data distribution, appropriate statistical tests were applied. For within-group comparisons between preoperative and six-month postoperative values, a paired Student’s t-test was used for normally distributed data, while the Wilcoxon signed-rank test was applied for non-normally distributed data. For between-group comparisons of independent samples, an independent Student’s t-test was performed for parametric data, and the Mann–Whitney U test was used for non-parametric data. A *p*-value of less than 0.05 was considered statistically significant for all analyses. Where necessary, adjustments for multiple comparisons were applied using the false discovery rate (FDR) method to minimize the risk of Type I errors. To account for the potential correlation between the two eyes of the same patient, generalized estimating equations (GEE) were used with an exchangeable correlation structure. This approach ensures that intra-subject dependencies are properly adjusted, preventing statistical bias while allowing the inclusion of both eyes in the analysis. The model estimates were obtained using robust standard errors to provide reliable inference.

## 3. Results

A total of 30 eyes from 20 patients were included in the study. The age ranged from 18 to 51 years, with a mean age of 33.87 years (SD = 9.46). Regarding sex distribution, 70% of the patients (*n* = 21) were male, while 30% (*n* = 9) were female. Table 1 summarizes the refractive and visual acuity outcomes before and after the TE-ACXL procedure. A statistically significant improvement was observed in UDVA and CDVA, with an increase in decimal notation values and a corresponding reduction in LogMAR values. Refractive parameters also showed changes, with a notable decrease in the spherical and cylindrical components. The UDVA in decimal notation increased by 0.16 ± 0.18, while the corresponding LogMAR UDVA decreased by 0.18 ± 0.21. CDVA in decimal notation showed an increment of 0.19 ± 0.21, whereas its LogMAR counterpart exhibited a decrease of 0.14 ± 0.19. Refractive parameters exhibited modifications, with the spherical component decreasing by −0.87 ± 2.27 D and the cylindrical component reduced by −1.00 ± 1.90 D.

Table 2 presents the topographic and tomographic parameters before and after the procedure. Maximum keratometry exhibited a significant reduction, suggesting a flattening effect on the cornea. Maximum keratometry decreased by 1.14 ± 0.69 D. Corneal thickness parameters, including TCT and CCT, also showed a statistically significant decrease. The TCT decreased by 27.80 ± 10.89 µm, and the CCT decreased by 29.56 ± 21.87 µm.

**Figure 1:**
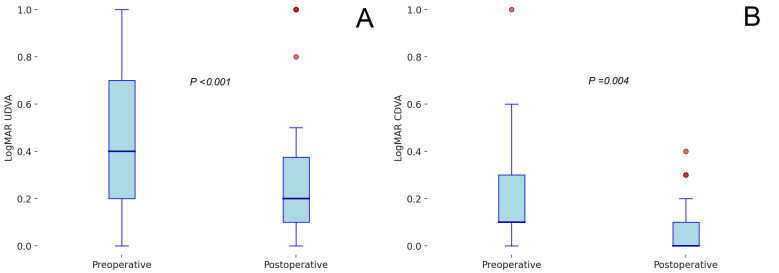

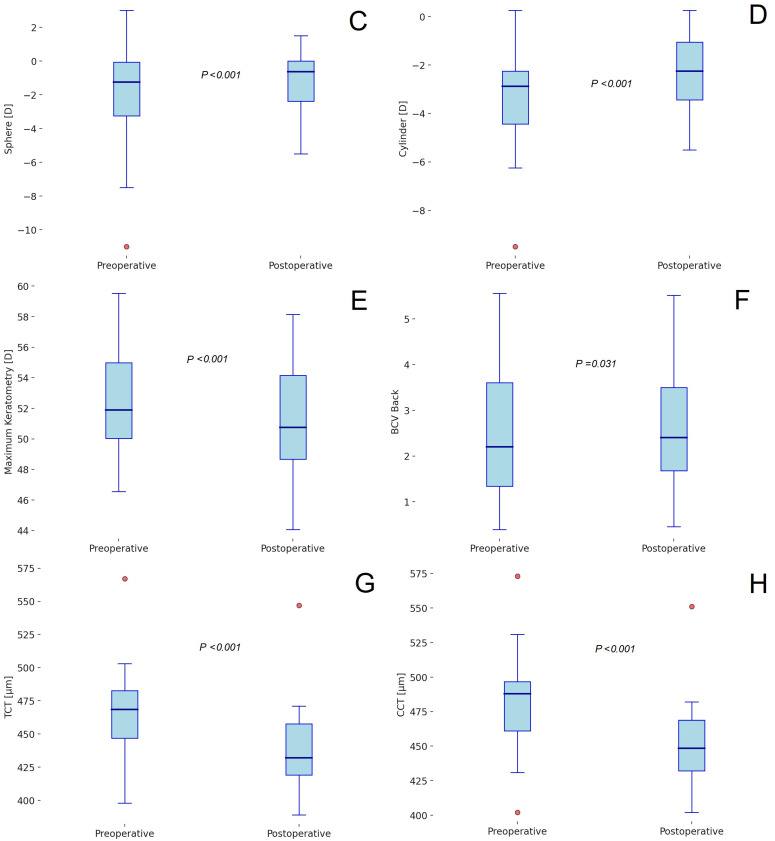
Box plots of preoperative and postoperative visual and corneal parameters. (**A**) Logarithm of the minimum angle of resolution (LogMAR) uncorrected distance visual acuity (UDVA), (**B**) logarithm of the minimum angle of resolution (LogMAR) corrected distance visual acuity (CDVA), (**C**) spherical refractive error (sphere, in diopters [D]), (**D**) cylindrical refractive error (cylinder, in diopters [D]), (**E**) maximum keratometry (max K, in diopters [D]), (**F**) back Baiocchi–Calossi–Versaci index (BCV back), (**G**) thinnest corneal thickness (TCT, in micrometers [µm]), and (**H**) central corneal thickness (CCT, in micrometers [µm]). Colored dots are outliers.

Corneal symmetry indices displayed minimal variation, with the Si front showing a negligible decrease of 0.01 ± 1.05 and the Si back experiencing a slight increase of 0.03 ± 0.16. The KV front decreased by 0.26 ± 4.46, while the KV back demonstrated an increment of −2.00 ± 7.21. The Baiocchi–Calossi–Versaci index showed minor changes, with the front component increasing by −0.01 ± 0.44 and the back component increasing by −0.12 ± 0.35. Figure 1 presents box plots comparing preoperative and postoperative measurements of visual acuity, refractive error, and corneal parameters. Together, these findings indicate that TE-ACXL leads to improvements in visual acuity and refractive outcomes, alongside measurable modifications in corneal biomechanics and topography. No statistically significant differences were observed between male and female patients in terms of visual acuity or tomographic outcomes (*p* > 0.05), indicating that gender did not influence the treatment response in this cohort.

## 4. Discussion

This study demonstrates significant postoperative improvements in both uncorrected and corrected distance visual acuity, with a notable reduction in spherical and cylindrical refractive errors. Additionally, corneal parameters such as Kmax, BCV back, TCT, and CCT showed measurable changes, indicating structural remodeling and optical optimization following the intervention. These findings highlight the efficacy of the procedure in enhancing both visual function and corneal stability.

### 4.1. Visual Acuity and Refractive Improvements

The significant improvements in CDVA observed in our study are consistent with previous reports on CXL efficacy. Vidas et al. [15] demonstrated similar visual acuity gains with both conventional (CXL30) and accelerated (CXL10) epi-off CXL protocols, with the conventional approach inducing more significant structural changes. Our results similarly show improved visual acuity and corneal stabilization, though the degree of remodeling may depend on treatment parameters and patient characteristics.

Our study also aligns with Cronin et al. [1,16], who investigated customized topography-guided TE-CXL with oxygen supplementation, reporting sustained improvements in CDVA and keratometry. While their study emphasized the benefits of tailoring UV-A exposure based on corneal topography, our standardized protocol yielded comparable improvements in visual function and refractive outcomes.

Similar findings have been reported by Kamiya et al. [13], who observed a significant reduction in refractive cylinder and improvements in both uncorrected and best-corrected visual acuity following oxygen-supplemented, customized TE-CXL. Additionally, Hamida et al. [17] found that the degree of CDVA improvement varied based on preoperative corneal thickness and higher-order aberrations, suggesting that patient-specific factors influence visual recovery after CXL.

### 4.2. Corneal Remodeling and Stability

The observed changes in Kmax, thinnest corneal thickness, and BCV back in our study are consistent with reports by Mazzotta et al. [9,17,18,19], who investigated various accelerated CXL protocols, including pulsed-light and high-fluence approaches. Their studies demonstrated significant corneal stiffening and topographic stabilization, comparable to our findings and with demarcation lines averaging between 250 and 280 µm. The structural remodeling we observed suggests that CXL slightly enhances corneal biomechanical stability, even in cases of moderate-to-advanced keratoconus.

Similar structural stability has been reported by Hill et al. [20], who examined the impact of supplemental oxygen in TE-CXL, finding that increased stromal oxygenation enhances cross-linking efficacy. Our results, which demonstrate significant corneal thickening and stabilization, further support the hypothesis that oxygen availability plays a critical role in optimizing CXL outcomes.

Further comparisons can be drawn with Caruso et al. [21], who evaluated the conventional Dresden protocol versus accelerated custom-fast CXL, finding no significant differences in visual and refractive outcomes between protocols. Our findings similarly suggest that accelerated protocols can achieve comparable stabilization to conventional CXL while minimizing treatment duration.

### 4.3. Efficacy in Advanced and Pediatric Keratoconus

Our study supports the efficacy of TE-ACXL in improving corneal stability and visual function, similar to findings by Sun et al. [14], who assessed transepithelial accelerated CXL in advanced keratoconus (Kmax > 58 D). While their study found comparable efficacy across different severity levels, our data suggest that the extent of refractive improvement may depend on additional factors such as corneal thickness and topographic irregularities.

Similarly, Abdel-Radi et al. [22] found that TE-ACXL prevented disease progression in tomographically suspicious fellow eyes of young patients with unilateral keratoconus, compared with observation alone, as evidenced by a significantly lower progression rate in the TE-ACXL group (4.2%) versus the observation group (42.1%) over a 24-month follow-up period. Our results, which indicate significant structural stabilization, align with their findings, and further support the role of early intervention in at-risk cases.

### 4.4. Long-Term Considerations and Progression Risks

While our study confirms the short-to-medium-term benefits of CXL, the long-term stability of these improvements remains a concern. Vilares-Morgado et al. [2] reported a high rate of keratoconus progression (over 60%) within four years following TE-CXL, with 26.8% of eyes requiring retreatment. Although our study shows promising structural changes, future follow-up is necessary to determine whether these improvements remain stable over extended periods. This aligns with findings from Yuksel et al. [10], who observed that epi-off CXL resulted in greater keratometric flattening compared to TE-CXL over 30 months. While our results demonstrate significant improvements in visual acuity and corneal parameters, the potential for continued progression necessitates further monitoring.

Vilares-Morgado et al. [2] reported that TE-ACXL is associated with lower postoperative discomfort compared to epithelium-off protocols, mainly due to epithelial preservation. Similarly, Yuksel et al. [10] observed reduced pain levels in TE-ACXL patients, though mild transient haze and discomfort were noted in some cases. Nicula et al. [23] highlighted that variations in riboflavin formulations and UVA parameters may influence postoperative discomfort, despite the advantages of epithelial preservation.

### 4.5. Limitations

This study has several limitations that should be considered when interpreting the results. First, the retrospective design introduces potential selection bias, as patient inclusion was dependent on the availability of complete follow-up data rather than a randomized selection process. A prospective study design would provide more controlled data acquisition and reduce confounding factors. Second, the sample size was small, which may limit the statistical power and generalizability of the findings. A larger cohort would be necessary to confirm these results and to allow for subgroup analyses based on different severities of keratoconus. A limitation of this study is the absence of a control group, as all included patients underwent TE-ACXL. Given the ethical considerations of leaving progressive keratoconus untreated, a control group was not feasible. However, future studies could incorporate comparative groups or historical controls to further validate these findings.

Third, the follow-up period was limited to six months, which may not be sufficient to fully assess the long-term stability of the corneal changes induced by TE-ACXL. Corneal and refractive parameters continue to evolve beyond six months, making longer follow-up studies essential for evaluating sustained efficacy and safety. Fourth, the study lacks a control group, preventing direct comparison with untreated eyes or with eyes that underwent alternative crosslinking protocols such as standard epithelium-off (epi-off) CXL. The inclusion of a control group in future studies would strengthen the validity of the conclusions regarding the efficacy of TE-ACXL.

Fourth, the absence of fluorescein staining in the evaluation protocol. Although all patients underwent slit-lamp biomicroscopy, fluorescein staining could have provided additional information on corneal epithelial integrity. Future studies should consider incorporating this assessment for a more comprehensive evaluation.

Finally, while improvements were observed in visual acuity and refractive parameters, corneal topographic indices showed only minor, non-statistically significant changes at the six-month follow-up. This raises questions about the biomechanical impact of TE-ACXL and suggests that additional biomechanical assessments may be needed to fully understand the structural effects of the procedure.

### 4.6. Future Lines of Research

Future research on TE-ACXL should focus on optimizing treatment efficacy and personalization through novel technologies. One promising direction involves enhancing riboflavin delivery by developing nanocarrier-based formulations, such as liposomal or nanoparticle-enhanced riboflavin, which could improve stromal penetration while preserving the epithelium, potentially leading to more effective crosslinking. Another key area is the integration of artificial intelligence and machine learning to optimize treatment parameters. AI-driven predictive models could analyze topographic and biomechanical data to tailor UV exposure and riboflavin dosing on an individual basis. Additionally, the development of wearable or remote monitoring technologies could revolutionize keratoconus management. The use of tear film biomarkers, corneal biomechanics sensors, or smart contact lenses could enable continuous real-world tracking of keratoconus progression, allowing for earlier intervention and improved long-term outcomes.

### 4.7. Clinical Implications

The observed improvements support the role of TE-ACXL as an effective minimally invasive treatment option. By preserving the corneal epithelium, this technique reduces postoperative discomfort and lowers the risk of infection and haze, making it particularly suitable for patients who may not tolerate the conventional epi-off approach. The stabilization of corneal parameters observed at six months suggests that TE-ACXL provides structural reinforcement, helping to slow or halt the progression of keratoconus. This is clinically relevant, as early intervention with TE-ACXL could delay or potentially eliminate the need for more invasive surgical procedures, such as intracorneal ring implantation or corneal transplantation. These results reinforce the importance of regular monitoring of keratoconus progression and highlight the need for personalized treatment strategies.

## 5. Conclusions

This study demonstrates that TE-ACXL is a safe and effective treatment for progressive keratoconus, leading to improvements in visual acuity and refractive stability at six months postoperatively. The preservation of the epithelium contributes to reduced postoperative discomfort and a lower risk of complications, making it a favorable alternative to conventional epi-off protocols.

The findings suggest that TE-ACXL may help stabilize keratoconus progression in the short term, with positive effects on visual and corneal parameters. These results reinforce their role as a minimally invasive approach with a good safety profile, offering a treatment option for patients seeking a less disruptive recovery while aiming to slow the natural course of the disease.

## Figures and Tables

**Table 1:** Refractive and visual acuity outcomes before and after TE-ACXL.

Variables [Units]	Preoperative Data	Postoperative Data	*p*-Value
Sphere [D]	−2.18 ± 3.05 (−11.00 to 3.00)	−1.31 ± 1.84 (−5.50 to 1.50)	<0.001
Cylinder [D]	−3.33 ± 1.98 (−9.50 to 0.25)	−2.33 ± 1.52 (−5.50 to 0.25)	<0.001
UDVA [decimal]	0.44 ± 0.30 (0.05 to 1.00)	0.61 ± 0.29 (0.05 to 1.00)	<0.001
UDVA [LogMAR]	0.47 ± 0.35 (0.00 to 1.00)	0.29 ± 0.30 (0.00 to 1.00)	<0.001
CDVA [decimal]	0.71 ± 0.25 (0.10 to 1.00)	0.90 ± 0.17 (0.40 to 1.00)	0.022
CDVA [LogMAR]	0.20 ± 0.22 (0.00 to 1.00)	0.06 ± 0.11 (0.00 to 0.40)	0.004

UDVA: Uncorrected distance visual acuity; CDVA: corrected distance visual acuity; LogMAR: logarithm of the minimum angle of resolution.

**Table 2:** Topographic and tomographic parameters before and after TE-ACXL.

Variables [Units]	Preoperative Data	Postoperative Data	*p*-Value
Kmax [D]	52.33 ± 3.51 (46.55 to 59.52)	51.19 ± 3.63 (44.06 to 58.14)	<0.001
Si front	5.38 ± 3.01 (0.37 to 12.44)	5.37 ± 2.75 (0.28 to 12.01)	0.479
Si back	1.43 ± 0.74 (0.29 to 2.78)	1.46 ± 0.69 (0.33 to 2.67)	0.153
KV front	24.43 ± 13.41 (4 to 55)	24.17 ± 11.53 (4 to 48)	0.373
KV back	53.53 ± 30.10 (8 to 128)	55.53 ± 30.62 (9 to 136)	0.070
BCV front	2.55 ± 1.40 (0.36 to 5.67)	2.56 ± 1.26 (0.37 to 5.03)	0.420
BCV back	2.52 ± 1.43 (0.39 to 5.56)	2.64 ± 1.36 (0.46 to 5.52)	0.031
TCT [µm]	466.43 ± 31.24 (398 to 567)	438.63 ± 30.54 (389 to 547)	<0.001
CCT [µm]	480.80 ± 33.24 (402 to 573)	451.23 ± 29.26 (402 to 551)	<0.001

Kmax: maximum keratometry (steepest corneal curvature); Si: symmetry index of the front and back corneal surface; KV: keratoconus vertex of the corneal surface (front and back); BCV: Baiocchi–Calossi–Versaci index of the corneal surface (front and back); TCT: thinnest corneal thickness; CCT: central corneal thickness.

## Data Availability

The raw data supporting the conclusions of this article will be made available by the authors on request.

## Abbreviations

The following abbreviations are used in this manuscript:

BAC	Benzalkonium Chloride
BCV	Baiocchi–Calossi–Versaci Index
BSS	Balanced Salt Solution
CCT	Central Corneal Thickness
CDVA	Corrected Distance Visual Acuity
CXL	Corneal Collagen Crosslinking
EDTA	Ethylenediaminetetraacetic Acid
HOA RMS	Higher-Order Aberrations Root Mean Square
KV	Keratoconus Vertex
Kmax	Maximum Keratometry
LogMAR	Logarithm of the Minimum Angle of Resolution
PRC	Posterior Radius of Curvature
Si	Symmetry Index
TCT	Thinnest Corneal Thickness
TE-ACXL	Transepithelial Accelerated Corneal Crosslinking
TE-CXL	Transepithelial Corneal Crosslinking
Tris	Trometamol
UDVA	Uncorrected Distance Visual Acuity
UV-A	Ultraviolet-A
VE-TPGS	Vitamin E TPGS (D-α-Tocopheryl Polyethylene Glycol-1000 Succinate)
aCFXL	Accelerated Custom-Fast Corneal Crosslinking
epi-off CXL	Epithelium-Off Corneal Crosslinking
epi-on CXL	Epithelium-On Corneal Crosslinking
sCXL	Standard Dresden Protocol Corneal Crosslinking
